# Asperger Syndrome (AS): A Review Article

**DOI:** 10.7759/cureus.31395

**Published:** 2022-11-11

**Authors:** Vidhi Motlani, Gunjan Motlani, Archana Thool

**Affiliations:** 1 Medical Education, Jawaharlal Nehru Medical College, Datta Meghe Institute of Medical Sciences, Wardha, IND; 2 Ophthalmology, Jawaharlal Nehru Medical College, Datta Meghe Institute of Medical Sciences, Wardha, IND

**Keywords:** treatment, intellectual disability, neuropsychiatric disorders, cognitive impairment, autism spectrum disorders

## Abstract

A variant of autism spectrum disorder (ASD) known as Asperger syndrome (AS) is characterized by severe issues with interpersonal, verbal, and nonverbal communication as well as restricted and repetitive patterns of behavior and activities. Although there is no known cause for ASD, various genetic as well as non-genetic risk factors that may act either alone or in combination to induce ASD have been identified. The occurrence of ASD has been increasing worldwide. Co-occurring neuropsychological diseases are frequently present as well. The premise for diagnosis is the observation of abnormal conduct, with diagnostic criteria emphasizing limitations in social interaction and communication as well as constrained, repetitive behavioral patterns, activities, or hobbies. The degree of the illness and the presence of intellectual impairment have a significant impact on the prognosis. Like autism, Asperger's can neither be prevented nor treated. There is no specific medical therapy that can effectively cure all of the symptoms of autism. However, medications may be used as adjuvant therapy for maladaptive behaviors and co-occurring mental problems. The treatment aims to reduce core impairments, increase functional ability, and reduce harmful behaviors that could limit functional skills. It is crucial to provide proper care, establish supportive networks for individuals who are affected and their families, and use effective therapies to enhance functioning and results.

## Introduction and background

Asperger syndrome (AS) is a part of a broader category called autism spectrum disorder (ASD) [1]. It is a type of neuro-developmental disorder characterized by difficulty interacting with others, impairment in communication skills, and confined, repetitive, and stereotyped behavioral patterns, activities, and interests [2]. Asperger’s patients grow a variety of distinctive traits. Although the causes of AS are complicated and yet relatively unknown, hereditary and environmental factors are more likely to be involved [3]. The variability of presenting clinical symptoms is a hallmark feature associated with medical and neuropsychiatric conditions [4]. AS very often co-occurs with other neuropsychiatric problems such as obsessive-compulsive behavior, sleep disorder, Tourette syndrome, Attention Deficit Hyperactivity Disorder, and anxiety disorder [2]. It makes people more likely to commit crimes, impairs their intellectual ability and sense of responsibility, and hinders their capability for testifying or participating in legal proceedings [5]. AS is thought to affect between 0.02% and 0.03% of children. AS is less likely in girls as compared to boys, with an 8:1 sex ratio. There are presently no comprehensive investigations of the occurrence in adults. Patients with AS may display atypical social behavior and communication difficulties, which have an impact on their social and professional lives. It is also important to note that the key signs and symptoms of AS persist throughout the life of the patient [1]. Patients with AS are also generally preoccupied with maintaining the stability of their external surroundings and regular habits; sudden shifts may be too much for them to handle [1]. The family of a child with AS is more likely to confront significant levels of stress, due to the characteristics of the disease and the maladaptive behaviors that these children with AS display [6]. Children and adolescents with high-functioning autism (AS) present with a “triad” of impairments, including communication challenges, stereotypical/repetitive behaviors, and interests, and social problems [7]. 

Communication difficulties

Children with AS may face challenges in acquiring language skills and comprehending what people are saying to them. They frequently struggle with nonverbal cues including facial expressions, eye contact, and hand movements as well. Language and communication skills in children with Asperger syndrome are affected by their intellectual and social development [8].

Social disturbances

The lack of an instinctive understanding of social norms is the key characteristic. From an early age, the affected individual stands out as being socially secluded and showing minimal interest in making or keeping friends, especially with peers their own age. The existing forms of social interaction could be bizarre or incredibly self-centered [9].

Emotional and behavioral symptoms

Some of the emotional and behavioral symptoms include resistance to change, rigid adherence to routines, repetitive and stereotyped behavior, abnormal response to sensory stimuli, exaggerated emotional responses, regulatory attention disturbances, and unusual eating habits [8,9].

Etiology

AS is significantly heterogeneous in its etiology. AS etiology has been linked to a variety of genetic, neurological, and environmental variables, but the precise underlying mechanisms are still poorly understood [10].

Genetic Factors 

Genomic sequencing data suggests that hundreds of genes are associated with the disorder, Given the fact that no single gene alteration has been identified as being specific to AS. AS-related genes are engaged in a wide range of biological processes that affect the maturation and functioning of the brain [8].

Environmental Factors

Environmental factors, such as obstetric event, perinatal age, parental age and maternal factors, fetal environment, and exposure to toxic substances and teratogens, may serve as independent important risk factors or may impact already-existing genetic factors in those with a genetic susceptibility [4,8].

Diagnosis

A thorough assessment should be conducted as part of an AS evaluation, ideally by an interdisciplinary team. The evaluation's objectives are to conclusively diagnose AS, rule out conditions that mimic AS, recognize co-morbid conditions, and establish the child's functional level [11]. The role of ASD team in evaluation of children with AS is elaborated in Table 1.

**Table 1:** Interdisciplinary assessment team for children with autism spectrum disorder (ASD) Source: Sanchack and Thomas [11]

TEAM MEMBER	ROLE
Audiologist	Evaluates for hearing loss as contributor to developmental delay
Psychiatrist	Evaluates and treats associated psychiatric conditions and maladaptive behavior
Speech language pathologist	Evaluates for expressive receptive and programmatic language deficit, develops plan for treatment
Geneticist and genetic counselor	Performs evaluation when an underlying medical condition or genetic syndrome is suggested by family history, examination or clinical course, counsels family on recurrence rate
Developmental pediatrician, child neurologist, physician	Perform medical evaluation, identify and treat associated conditions
Occupational therapist	Evaluates for fine and gross motor deficits and sensory processing deficits, develops plans for treatment
Social worker	Identifies family needs, refers family to formal and informal support agencies and organizations

## Review

Alternative therapies, management, and treatment of AS

Early identification and treatment outcomes of these comorbid psychiatric illnesses are castigatory [2]. It is crucial to provide proper care, establish networks of support for afflicted people and their families, and use effective therapies to enhance functioning and results [4]. Asperger's contribution to the area extended beyond simply recognizing and characterizing this illness; he also worked to raise awareness of autism because he was worried that children with it would be misdiagnosed and mistreated. Additionally, he promoted an educational strategy that focused on giving each student particular attention, highlighting their strengths rather than their faults, and encouraging learning by leveraging their unique interests [12]. If not treated earlier, then it may lead a person to encounter extreme difficulty in social interactions, which may eventually cause anxiety to develop [6].

Treatment for AS includes medication as well as non-medication [13].

Pharmacological therapies

Difficulties associated with AS are no longer treated with standardized procedures or a specific pharmacological prescription, although some drugs can alleviate symptoms like irritability, depression, aggression, anxiety, or hyperactivity [13].

Risperidone

The Food and Drug Administration (FDA) approved risperidone, a second-generation antipsychotic, as the first medication to treat irritability linked to autism. This also proves that risperidone works well for treating behavioral issues and AS-associated symptoms in children [14].

Aripiprazole

It is a psychotropic drug. It is used to treat irritability in children and, in addition, is also indicated for the management of Bipolar disorder, Tourette syndrome, schizophrenia, and depression.

Selective serotonin reuptake inhibitors (SSRIs)

Drugs including sertraline, fluoxetine, and citalopram are used to reduce repetitive behaviors in AS.

Oxytocin

It has been discovered that this endogenous hormone plays a significant role in the development of relationships and social behavior in humans. These medications should be offered in accordance with each patient's specific needs, taking into account both the potential advantages and risks [14].

Non-pharmacological therapies

Cognitive Behavioral Therapy (CBT)

CBT intervention includes five main components: (a) framework; (b) group environment; (c) psycho-education, such as presentations and conversations on Asperger's and psychiatric conditions, as well as instruction on how to recognize and critically assess self-defeating thoughts; (d) developing social skills, such as making practiced phone calls and asking for assistance, and (e) cognitive behavioral strategies, such as goal-setting, job, exposure training, and behavior analysis [15]. CBT deals with the cognitive deficits and distortions that underlie many social and communication problems that lead to anxiety. CBT aims to assist the person to create a positive behavioral change and mood, identify these false belief patterns, and change them. Through CBT, individuals receive techniques to alter their thoughts and beliefs as well as necessary abilities (such as the ability to properly communicate with others and solve problems) [6].

Language and Speech Therapy 

The main diagnostic standards for AS are speech and language deficits. As a result, language skills can be nonverbal or extremely idiosyncratic, including echolalia (repetition of speech by a child learning to talk) and abnormal prosody (inflection or tone) [16]. Language delay and language-based deficits management focus mainly on pronunciation and the development of active or passive vocabulary, as well as on the phonetic-phonological level of language [17].

Learning and Writing Skills

The self-regulated strategy development (SRSD) is currently being used to treat children with educational/learning difficulties. Results of the strategy show that when children experiencing learning challenges are taught self-control and writing techniques (self-monitoring, goal setting, and self-reinforcement) procedures, both the quality as well as quantity of their writing gets improved. Instruction in SRSD offers students with different approach such as writing, editing, planning, revising and monitoring their own activities [18]. 

*Social Skills Training* *(SST)*

One of the most popular interventions to help with social difficulties in people with high-functioning autism (AS) is SST . Conventional SST gives face-to-face, in-person education on communication, companionship, and problem-solving abilities to individuals with disabilities in order to teach them how to engage with peers. Behavioral intervention technologies (BITs) are the emerging techniques used under SST delivery that are intended at eliciting beneficial psychological and behavioral changes as an addition to or a replacement for face-to-face interventions [19]. One of the most successful interventions for schoolchildren and elders with high-functioning autism is that which enables individuals to recognize and give importance to the motor development underneath their thoughts and emotions. Other appropriate strategies for learning the skills are based on mirror neuron theory [20].

Mind-Body Therapies (MBTs)

About 30% of individuals with AS employ mind-body practices or therapies as an alternative. A broad number of techniques are included in MBTs, which place an emphasis on the relationship between the mind, body, as well as health. It has been demonstrated in the context that mind-body treatments can relieve the clinical manifestations of stress, anxiety, sleep issues, and depression. However, it is uncertain that people with AS may utilize mind-body treatments. Furthermore, it is unknown how effective these treatments are for AS patients [21]. There is conflicting evidence about whether or not the theory of mind development is impaired in individuals with AS [22].

Family Support

AS brings extraordinary intricacy in a family. Parents, spouses, and relatives might require professional therapy or group therapy, especially if they too struggle with communicating, have remarkable perspective, or are persistent with their goals [5]. The degree of criticism from their parents was significantly associated with the autistic child's behavior issues [23]. As compared to the female parent of the normal growing children, parents of child with neuropsychiatric illnesses encounters more psychiatric issues, stress, anxiety, and poorer health [24]. Parents who are under a lot of stress are less capable of performing therapies for their disabled children, and their children develop more slowly as a result. However, the welfare of the child is the main focus of programs for families of children with impairments. Parental interventions comprise supportive care for the child, creating opportunities for socializing, participation in organizations for individuals with disabilities, advocacy teaching, and structuring the child's daily routines. [24].

Physical Therapy

It suggests that practicing multisensory enhanced yoga can benefit an AS sufferer and their off-the-mat behaviors relating to their physical, emotional, and social well-being [25].

Fecal Microbiota Transplantation (FMT) as a Treatment in AS

The human gut and central nervous system interact in complex ways in both directions through the gut-brain axis (GBA), which links the brain's cognitive and emotional regions to the peripheral functions of the intestine, and abnormal conditions in the gut may predispose individuals to various neurodevelopmental disorders [26]. Psychological disorders have been demonstrated to be impacted by gut microbiota-targeted therapies. FMT is a new treatment that involves repopulating the patient's gastrointestinal tract with fecal microbiota obtained from healthy individuals in order to reestablish microbial balance and rebuild the patient's gut microbiota [27]. Altering the gut microflora is potential way to assist in reducing gastrointestinal tract symptoms as well as behavioral problems in a child with AS [28].

Occupational Therapies

Many people with AS have impairments in their fine motor control, especially their graphomotor skill sets and level of automation, and also their motor planning [29]. Enhancing health and involvement in society through occupation is a focus of the occupational therapy profession. Various fields of occupation are covered by the occupational therapy practice framework (OTPF), which provides a basis for occupational therapy interventions. social interaction, education, and play are three of a student's main OTPF regions of occupation [30].

*Sensory-Based Interventions* 

Sensory input from the tactile, proprioceptive, and vestibular systems was used most frequently in intervention studies. It was discovered that the main goal of the therapy was to help patients learn how to pay attention, behave, process sensory information, as well as engage in a natural setting [30]. Early children's social training programs have received a lot of support, but a thorough independent assessment must be carried out. Despite the continuation of the underlying neurological abnormalities, the social development of an autistic kid can be expedited and occasionally returned to its ordinary course if a connection can be established [31]. For children under the age of five, which is the essential age for an AS diagnosis, families typically seek pediatricians and family physicians first. There may have been a delay in diagnosis because doctors were worried about the parents' concern about their child having AS. They may have feared the label would have detrimental effects on the individuals. Additionally, some medical experts think they lack the necessary training to assess AS [32]. Also health officials, autism advocacy groups, and many other stakeholders use social media platforms to spread information and awareness on AS and autism [33]. Virtual reality (VR), a device-based simulation of reality wherein visual representations dependent on ordinary life situations are exhibited on a screen, is one platform that offers an opportunity to practice dynamic and real-world social interaction. VR has been tested and proven to be a successful intervention technique for a number of disorders, including anxiousness, phobias, fear of flying, strokes, and post-traumatic trauma stress disorders [34]. It has been demonstrated that community-based programs with a strong emphasis on leisure enhance participants' quality of life and independence. In a similar manner, participants in programs emphasizing supported employment, with job assistance, a job coach, collaboration with the individual's greater social support network, and great variety of activities to match a participant's strengths and abilities showed improved learning ability, particularly executive functioning, and employment [35].

## Conclusions

AS is not a distinct disorder but a milder variant of autism. Individuals with AS are also at higher risk for certain psychiatric and medical disorders, such as anxiety, depression, and seizures. Characteristics of AS include repetitive routines or rituals, peculiarities in speech and language, socially and emotionally inappropriate behavior, and an inability to interact successfully with peers, problems with nonverbal communication, and clumsy and uncoordinated motor movements. AS has no cure but clinical features and those secondary to comorbid conditions could improve with an early diagnosis and correct individualized interventions. Timing and quality of tailored therapeutic strategies are essential for the prevention and management of maladaptive behaviors and social issues. The assessment of children and adolescents with AS is best conducted by an interdisciplinary team capable of covering the central developmental and symptomatic aspects of the condition.
